# *γ*-Aminobutyric acid enhances myogenesis and heat/cold stress resistance in bovine muscle satellite cells

**DOI:** 10.3389/fvets.2026.1770540

**Published:** 2026-04-14

**Authors:** Abid Manzoor, Sajida Naseem, Zhiqi Fu, Chaohui Ruan, Xu Liu, Chunri Yan, Seongho Choi, Xiangzi Li

**Affiliations:** 1 Engineering Research Center of North-East Cold Region Beef Cattle Science & Technology Innovation, Ministry of Education, Department of Animal Science, Yanbian University, Yanji, China; 2 Medical College, Yanbian University, Yanji, China; 3 Department of Animal Science, Chungbuk National University, Cheongju, Republic of Korea

**Keywords:** bovine muscle satellite cells, oxidative stress, proliferation and differentiation, transcriptomics, *γ*-aminobutyric acid

## Abstract

**Introduction:**

*γ*-Aminobutyric acid (GABA) is the principal inhibitory neurotransmitter in the central nervous system and is involved in the development of neural tissue as well as the regulation of its functions. Meanwhile, GABA has also been demonstrated to confer multiple physiological benefits, including alleviating stress and improving metabolic homeostasis. This study investigated GABA effects on proliferation, differentiation, and temperature stress protection of bovine skeletal muscle satellite cells (BSCs).

**Methods:**

BSCs were exposed to graded GABA concentrations for 24–96 h; based on CCK-8 and EdU assays, 0.5 and 1 mM were selected for mechanistic experiments (RT-qPCR, Western blot, immunofluorescence). We further evaluated GABA under heat (41 °C) and cold (4 °C) stress by measuring stress-related genes, antioxidant indices, Nrf2/Keap1/HO-1 pathway proteins, and by performing transcriptomic analysis.

**Results:**

GABA significantly promoted BSC proliferation (*p* < 0.05) by upregulating *PCNA*, *Ki-67*, *CCND1*, *Pax3*, and *CDK2* mRNA, and enhanced myogenic differentiation (*p* < 0.05) by increasing MyoG, MyoD, and MyHC protein levels, with generally stronger effects at 0.5 mM. Under 41 °C, GABA reduced *Hsp72*, *HSP-H1*, *HSP27*, and *HSP90* mRNA (*p* < 0.05), whereas at 4 °C it increased *RBM3*, *CIRBP*, and *UCP2* mRNA and lowered intracellular ROS (*p* < 0.05). At 41 °C, 0.5 mM GABA elevated HO-1 protein (*p* < 0.05); at 4 °C, it upregulated *Nrf2*, *HO-1*, and *NQO-1* mRNA, while HO-1 protein declined (*p* < 0.05). Transcriptomics revealed enrichment of the p53 and PI3K-Akt pathways at 41 °C and the p53 and FoxO pathways at 4 °C (key nodes: *CDKN1A*, *ERBB3*, *HSPB1*, *CDK2*, *ESR2*, *CCNG2*, *FDFT1*, *CCNB1*). In an *in vivo* rumen-protected GABA (RP-GABA) trial under different THI conditions, RP-GABA significantly increased body length at Temperature Humidity Index (THI) 45 and THI 63 (*p* < 0.05).

**Conclusion:**

Across timepoints and indices, 0.5 mM GABA provided protection comparable to or better than 1 mM, suggesting 0.5 mM as the recommended comprehensive dose *in vitro* and offering molecular targets for functional feeds to improve ruminant resilience to temperature stress.

## 1 Introduction

Bovine skeletal muscle satellite cells (BSCs) are multipotent stem cells that are essential for the growth, maintenance, and regeneration of bovine skeletal muscle. These cells typically remain quiescent between the basal lamina and sarcolemma of mature myofibers. Upon specific stimuli such as muscle injury, exercise, or growth signals, satellite cells become activated, proliferate, and differentiate into myoblasts, which then fuse to form new myofibers or repair existing ones (1). In ruminants, a core adverse effect of temperature stress is the disruption of redox homeostasis, leading to oxidative stress. Both heat and cold stress increase the intracellular burden of reactive oxygen species (ROS), disturb normal metabolism and cellular functions, and ultimately compromise meat production efficiency and tissue health (2). At the cell level, excessive ROS activates stress damage pathways (e.g., p38/MAPK), diverts satellite cells from self-renewal to differentiation with premature senescence, and weakens regenerative potential, thereby impairing proliferation and repair (3, 4). Therefore, identifying safe molecules that enhance BSC activity and alleviate oxidative stress under temperature stress is of practical and biological importance.

GABA can alleviate oxidative damage to some extent at animal level and accelerate structural and functional recovery after acute or chronic muscle injury (5). Multiple studies suggest that GABA acts via autocrine/paracrine routes within the local microenvironment to regulate the cell cycle, direct differentiation trajectories, and systemic antioxidant defenses. Exogenous GABA modulates myogenic processes in cell models, including proliferation, fusion, and differentiation marker expression, suggesting that myogenic cells can sense and respond to GABA during myogenesis and regeneration (6, 7). In C2C12 myoblasts, exogenous GABA has been reported to promote proliferation and myogenic factor expression while upregulating phase II antioxidant enzymes [e.g., heme oxygenase-1 (HO-1) and NAD(P)H quinone dehydrogenase 1 (NQO-1)], accompanied by lower ROS accumulation and attenuated GSH depletion (8, 9). *In vivo*, dietary GABA improves oxidative damage and accelerates recovery from muscle injury (10). Although previous studies have shown that GABA exerts biological functions in regulating cell proliferation, antioxidant defense, and tissue repair, related work has so far been focused mainly on murine C2C12 cells or non-ruminant species (7, 11), and the mechanisms of GABA action in bovine BSCs remain to be systematically defined. Temperature stress is a highly realistic stressor in ruminant production, there are few reports on whether GABA can enhance bovine BSCs survival and antioxidant defense system.

Yanbian Yellow cattle is an important indigenous Chinese beef breed with favorable meat quality (12). Enhancing their muscle growth potential is not only biologically meaningful but also directly relevant to genetic improvement and the sustainable development of local husbandry. Using BSCs from Yanbian Yellow cattle, we systematically assessed how GABA affects proliferative activity, myogenic differentiation, and antioxidant capacity and examined its regulation of key cell-cycle genes, myogenic regulators, and oxidative-stress pathways under simulated heat/cold stress. In addition, we included an *in vivo* rumen-protected GABA (RP-GABA) trial under different THI conditions to assess growth-related measures. The goal was to clarify GABA’s biological functions in BSCs and provide a theoretical basis for developing anti-stress functional feeds, improving beef-cattle growth performance, and protecting germplasm resources.

## 2 Materials and methods

All animal procedures were approved by the Institutional Animal Care and Use Committee of Yanbian University (Approval No. YD20240315007).

### 2.1 Isolation and culture of BSCs

Under sterile conditions, semimembranosus muscles were collected immediately after slaughter of Yanbian Yellow cattle. After removing visible connective tissue and fat from ~600 g fresh muscle tissue, the samples were washed three times with sterile PBS containing 3% penicillin-streptomycin (PS). Minced tissue was digested with 0.8% pronase at 37 °C for 1 h with gentle agitation every 10 min. After centrifugation at 1000×g , the cell pellet was washed with PBS containing 1% PS and resuspended in DMEM supplemented with 10% FBS and 3% PS. The suspension was passed through 100-μm and 70-μm filters successively, centrifuged at 700×g for 10 min, and resuspended in growth medium (DMEM + 10% FBS + 1% PS). Paired box protein 7 (PAX7), Desmin, and myogenin (MyoG) antibodies were used for BSCs identification through immunofluorescence assay.

### 2.2 Treatment of BSCs

BSCs of passage 3 were used for experiments. BSCs were plated in 6, 12, 24, or 96 h plate according to experimental need. When BSCs reached about 50% confluence, they were treated with different concentrations of GABA in growth medium (DMEM + 10% FBS + 1% PS) to evaluate its effects on BSC proliferation. For differentiation, when cells reached ~80% confluence, the growth medium was replaced with differentiation medium (DMEM + 2% horse serum + 1% penicillin-streptomycin), and cells were cultured for 2, 4, or 6 days. For heat- and cold-stress treatments, cells were pretreated with GABA for 48 h at 37 °C in 5% CO_2_ and then exposed to 41 °C or 4 °C for 6 h.

### 2.3 Cell viability assay and 5-ethynyl-2′-deoxyuridine

Cell viability was measured with a CCK-8 kit (Apexbio, Shanghai, China) following the manufacturer’s instructions. Briefly, BSCs were seeded at 5 × 10^3^ cells/well in 96-well plates and allowed to adhere overnight. Then cells were treated with 0.1-, 0.5-, 1-, 2-, 5-, and 10-mM GABA for 24, 48, 72, or 96 h. At each timepoint, 10 μL CCK-8 reagent with 100 μL serum-free DMEM was added per well. After incubation at 37 °C, 5% CO_2_ for 4 h, absorbance was read at 450 nm on a microplate reader (De Tie, HBS-1096A). Each condition had six technical replicates.

EdU staining was performed with a commercial kit (RIBOBIO, Guangzhou, China). BSCs were seeded at 5 × 10^3^ cells/well in 96-well plates, allowed to adhere overnight, and treated for 48 h with 0.5, 1 and 2 mM GABA, three replicates per group. Briefly, cells were then incubated for 2 h in 100 μL medium containing 50 μM EdU at 37 °C, 5% CO_2_, fixed with 4% paraformaldehyde for 30 min, permeabilized with 0.1% Triton X-100 for 10 min, and stained with Hoechst 33342 for 30 min in the dark. Images were acquired on an inverted fluorescence microscope (WF10× objective, Olympus, Japan).

### 2.4 Immunofluorescence assay

BSCs were cultured in 96 well plate and fixed at room temperature with 4% paraformaldehyde for 20 min. After washing with phosphate-buffered saline (PBS), the cells were permeabilized with 0.2% Triton X-100 (Sigma-Aldrich) for 30 min at room temperature and rinsed again with PBS. Polyclonal antibodies against myogenin (MYOG; BS-3550R, Bioss, Beijing, China), paired box protein 7 (Pax7; AB-528428, Abcam, United Kingdom) and desmin (BS-20702R, Bioss, Beijing, China) were each diluted 1:100 in PBS containing 5% bovine serum albumin (BSA) and incubated overnight at room temperature. Subsequently, horseradish peroxidase (HRP)-conjugated goat anti-mouse IgG (H + L) (A0216, Beyotime, Shanghai, China) or goat anti-rabbit IgG-HRP (BS-3550R, Bioss, Beijing, China) was diluted 1:200 in PBS with 5% BSA and incubated for 2 h at room temperature in the dark. Finally, nuclei were counterstained with Hoechst for 30 min in the dark, and images were acquired using a fluorescence microscope (WF10× objective, Olympus, Japan), and analyzed by Image J software.

### 2.5 Extraction of RNA and real-time quantitative PCR

Total RNA was extracted from treated BSCs using TRIzol reagent (Thermo Fisher Scientific, United States). RNA purity and integrity were assessed, and 1 μg RNA was reverse-transcribed using the FastKing One-Step RT Kit (Tiangen, China). RT-qPCR was performed with SuperReal PreMix Plus SYBR Green (Tiangen) on an Agilent Mx3000P system. Cycling: 95 °C for 15 min; then 40 cycles of 95 °C for 10 s, 55 °C for 30 s, 72 °C for 32 s. Target genes included: cyclin-dependent kinase 2 (*CDK2*), proliferating cell nuclear antigen (*PCNA*), marker of proliferation Ki-67 (*Ki-67*), cyclin D1 (*CCND1*), paired box protein 7 (*Pax7*), paired box protein 3 (*Pax3*), myogenic factor 5 (*Myf5*), myogenic differentiation 1 (*MyoD*), myogenic regulatory factor 4 (*MRF4*), myogenin (*MyoG*), myosin heavy chain (*MyHC*), heat shock factor 1 (*HSF1*), heat shock protein 72 (*Hsp72*), heat shock protein family H member 1 (*HSPH1*), heat shock protein 27 (*HSP27*), heat shock protein 90 (*HSP90*), RNA-binding motif protein 3 (*RBM3*), cold-inducible RNA-binding protein (*CIRBP*), uncoupling protein 2 (*UCP2*), nuclear factor erythroid 2–related factor 2 (*Nrf2*), heme oxygenase-1 (*HO-1*), NAD(P)H quinone dehydrogenase 1 (*NQO-1*), glutathione peroxidase-1 (*GPX-1*), and superoxide dismutase-1 (*SOD-1*), to evaluate proliferation, myogenic differentiation, and antioxidant status in BSCs. GAPDH served as the internal control. Relative expression was calculated by the 2^−ΔΔCt^ method. Primer sequences are listed in Supplementary Table S1.

### 2.6 Western blotting

Total protein was extracted with RIPA buffer (Beyotime, China) plus protease and phosphatase inhibitors. Protein concentration was determined using a Pierce BCA kit (Thermo Fisher Scientific, United States). Equal amounts of protein were separated by 12% SDS-PAGE and transferred to PVDF membranes (Merck, Germany). Membranes were blocked with quick blocking buffer with 1X TBST for 20 min and incubated overnight at 4 °C with Primary antibodies included paired box 7 (Pax7), cyclin-dependent kinase 1 (CDK1), cyclin-dependent kinase 2 (CDK2), cyclin-dependent kinase inhibitor 1A (P21), myogenin (MyoG), myogenic differentiation 1 (MyoD), myosin heavy chain (MyHC), Kelch-like ECH-associated protein 1 (Keap-1), nuclear factor erythroid 2-related factor 2 (Nrf2), heme oxygenase-1 (HO-1), and glutathione peroxidase 4 (Gpx-4) (1:1000). After wash, membranes were incubated with HRP-conjugated secondary antibodies (goat anti-rabbit IgG, 1:10000; goat anti-mouse IgG, 1:1000) for 2 h at room temperature, developed by chemiluminescence, and imaged (Azure 600, Azure Biosystems, United States). β-actin was used as loading control, and bands were measured by Image J software.

### 2.7 Live and dead cell staining

Calcein-AM and propidium iodide (PI) were used to stain live and dead cells. After heat and cold stress treatment, cells were washed with PBS and incubated with staining solution (prepared according to the manufacturer’s instructions: 1 μL calcein-AM + 1 μL PI + 1 mL assay buffer) for 30 min at 37 °C. Cells were imaged by fluorescence microscopy.

### 2.8 Antioxidant index and ROS detection

Commercial kits (Beyotime, Shanghai, China) were used to measure reactive oxygen species (ROS) staining, glutathione peroxidase (GPx) and superoxide dismutase (SOD) activities and malondialdehyde (MDA) levels.

### 2.9 Sample RNA extraction, library construction, and sequencing

Total RNA was extracted with TRIzol. Integrity was verified by 1% agarose electrophoresis; concentration was measured by NanoDrop at 260/280 nm (OD260/280 of 1.8–2.0 was required). mRNA was enriched using oligo (dT) magnetic beads and fragmented under controlled conditions. First- and second-strand cDNA were synthesized (dUTP replacing dTTP in second-strand reactions for strand specificity). Double-stranded cDNA underwent end repair, A-tailing, and adaptor ligation; non-ligated and self-ligated products were removed; and libraries were amplified by PCR with adaptor-complementary primers. Libraries were purified with magnetic beads, quantified by Qubit, and fragment size assessed by Qsep400. Qualified libraries were sequenced on an Illumina platform using PE150 to ensure coverage and depth sufficient for transcriptomics.

### 2.10 Differentially expressed gene screening and functional enrichment analysis

Raw read counts from RNA-seq were used as input for differential expression analysis with the R package DESeq2 (v1.38.0). After normalization of gene expression levels between treatment groups, significantly differentially expressed genes (DEGs) were defined using an adjusted *p* < 0.05 and |log_2_FoldChange| ≥ 0.585 as the screening thresholds. To investigate the biological functions of DEGs, we performed Gene Ontology (GO) functional enrichment and Kyoto Encyclopedia of Genes and Genomes (KEGG) pathway enrichment analyses using the R package cluster Profiler (v3.14.3). GO analysis covered three major categories: biological process (BP), cellular component (CC), and molecular function (MF). KEGG pathway analysis was used to identify major metabolic and signaling pathways involved in cellular responses to GABA treatment. An adjusted *p* < 0.05 was considered significant for enrichment. Results were visualized using ggplot2 and enrich plot.

### 2.11 Protein–protein interaction network and hub genes

DEGs from significantly enriched KEGG pathways were submitted to STRING^1^ to construct PPI networks. Hub genes were identified with the Degree metric using CytoHubba.

### 2.12 Co-expression network analysis

Co-expression networks were constructed using DEGs that mapped to significantly enriched KEGG pathways. Pairwise Pearson correlation coefficients among these DEGs were calculated with the Hmisc package in R, and only significantly correlated gene pairs (*p* < 0.05) were retained to assemble the final gene co-expression networks (13).

### 2.13 *In vivo* RP-GABA supplementation and THI monitoring

Fourteen healthy castrated Yanbian Yellow calves (initial body weight: 281.21 ± 41.68 kg) were randomly allocated to a control group (basal diet without RP-GABA) or an RP-GABA group (850 mg/kg body weight; *n* = 7/group). The total experimental period lasted 209 days. Calves were fed twice daily (07:00 and 16:00) with free access to water. The trial was conducted at Longjing Hailanjiang Animal Husbandry Company, LTD. Ambient temperature (°C) and relative humidity (%) were recorded at 07:00, 14:00, and 22:00 using an environmental monitor placed centrally in the shed (~1.5 m above ground). The temperature–humidity index (THI) was calculated using the NOAA (1976) equation: THI = *T* − (0.55–0.55 × RH) × (*T* − 58) (Supplementary Figure S1). Based on THI, animals were evaluated under three environmental stress conditions: THI 63 (extreme heat stress), THI 45 (mild heat stress), and THI 28 (mild cold stress) (defined by the mean THI during the corresponding phases). The composition and nutrient levels of the basal diet are shown in Supplementary Table S2.

### 2.14 *In vivo* growth performance assessment

Growth-related morphometrics were recorded under each THI condition, including body length, body height, hip width, hip bone, chest width, chest depth, cannon bone, ischium width, and heart girth.

### 2.15 Statistical data analysis

Data were organized in Excel 2021. One-way or two-way ANOVA with Tukey’s multiple comparisons was performed in GraphPad Prism 10.4.1. Results were expressed as mean ± SD, *p* < 0.05 was considered statistically significant. Graphs were generated in GraphPad Prism 10.4.1.

## 3 Results

### 3.1 Identification of bovine muscle satellite cells

**Figure 1:**
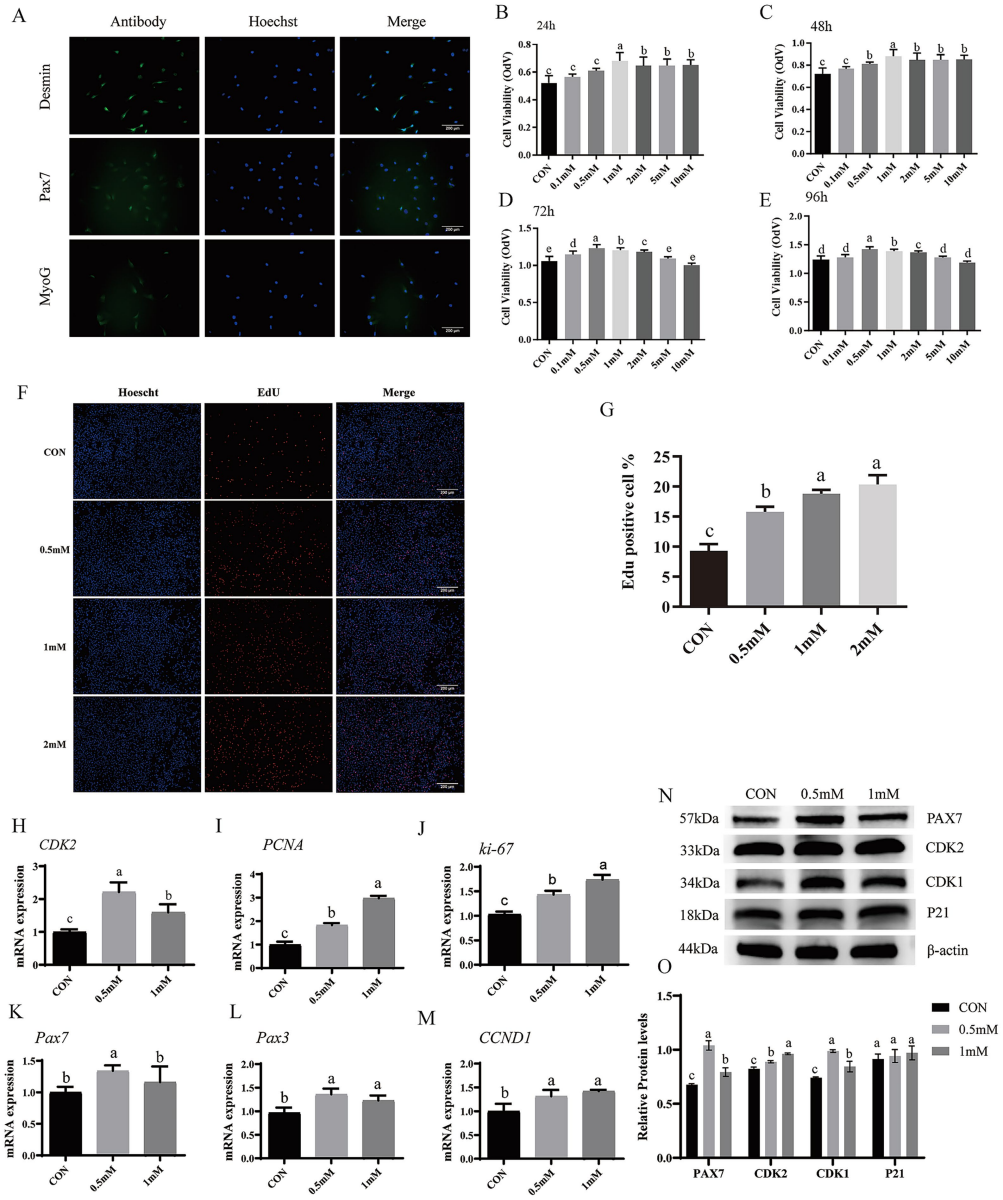
Effects of GABA on the proliferative activity and molecular markers in BSCs. **(A)** Immunofluorescence staining for BSCs identification (Desmin, Pax7, and MyoG (green) and nuclei (DAPI, blue) (10×; scale bar = 200 μm). **(B–E)** CCK-8 analysis of viability after 24, 48, 72, and 96 h treatment with 0.1, 0.5, 1, 2, 5, and 10 mM GABA and CON = 0 mM. **(F,G)** EdU assay. Hoechst (nuclei, blue) and EdU-positive cells (red) scale bar = 200 μm. **(H–M)** RT-qPCR. mRNA expression of proliferation-related genes (*CDK2*, *PCNA*, *Ki-67*, *Pax7*, *Pax3*, *CCND1*) after 48 h treatment with 0.5 and 1 mM GABA. **(N,O)** Western blotting of PAX7, CDK1, CDK2, and P21 proteins (β-actin loading control). (Mean ± SD; *n* = 3; groups with different letters a–e differs at *p* < 0.05).

BSCs were characterized by immunofluorescence staining for Desmin, PAX7, and MyoG (Figure 1A). Positive staining for these markers indicated successful isolation of bovine skeletal muscle satellite cells.

### 3.2 Effects of GABA on the proliferative activity of bovine muscle satellite cells

The CCK-8 assay assessed the effects of GABA on BSC proliferation (Figures 1B–E). A clear time- and dose-dependent response was observed. At 24 h the 1–10 mM GABA groups showed significantly increased viability as compared with the control group (*p* < 0.05), with the 1 mM group being significantly higher than the other groups (*p* < 0.05). At 48 h, compared with the control group the 0.5–10 mM treatment groups showed significantly increased viability (*p* < 0.05), highest with the 1 mM group (*p* < 0.05). At 72 h the 0.1–2 mM GABA groups showed significantly increased viability as compared with the control group (*p* < 0.05), with the 0.5 mM GABA group highest (*p* < 0.05). At 96 h, compared with the control group, the 0.5–2 mM groups showed significantly increased viability (*p* < 0.05), and highest in the 0.5 mM group (*p* < 0.05). At 72 h and 96 h, the 5 mM and 10 mM groups did not differ significantly from control (*p* > 0.05). Therefore, 0.5-, 1-, and 2-mM doses were selected for subsequent experiments.

### 3.3 Effects of GABA on EdU-labeled proliferative activity of bovine muscle satellite cells

EdU assays (Figures 1F,G) showed that, compared with the control group, all three GABA groups significantly increased the percentage of EdU positive cells (*p* < 0.05). The percentage of EdU positive cell were significantly increased by 1 mM and 2 mM GABA treatment than the 0.5 mM and control group (*p* < 0.05), while no significant difference between the 1 mM and 2 mM GABA groups (*p* > 0.05), indicating that increasing GABA concentration to 2 mM did not further enhance the proliferative effect.

### 3.4 Effects of GABA on expression of proliferation related genes and proteins in BSCs

We next measured mRNA expression of proliferation related genes (Figures 1H–M). The 0.5 mM and 1 mM group significantly upregulated *CDK2*, *PCNA*, *Ki-67*, *Pax3*, and *CCND1* mRNA (*p* < 0.05) as compared with the control group. Among them, *CDK2* and *Pax7* were significantly lower in the 1 mM group than in the 0.5 mM GABA group (*p* < 0.05), whereas *PCNA* and *Ki-67* were significantly higher in the 1 mM GABA group (*p* < 0.05). Furthermore, western blotting results (Figures 1N,O) showed that the PAX7, CDK2, and CDK1 proteins expression (*p* < 0.05) was significantly increased in 0.5 mM and 1 mM group as compared to control group. PAX7 and CDK1 were significantly higher in the 0.5 mM than in the 1 mM group (*p* < 0.05), whereas CDK2 was significantly lower in the 0.5 mM than in the 1 mM group (*p* < 0.05).

### 3.5 Effects of GABA on myogenic differentiation related genes and proteins

**Figure 2:**
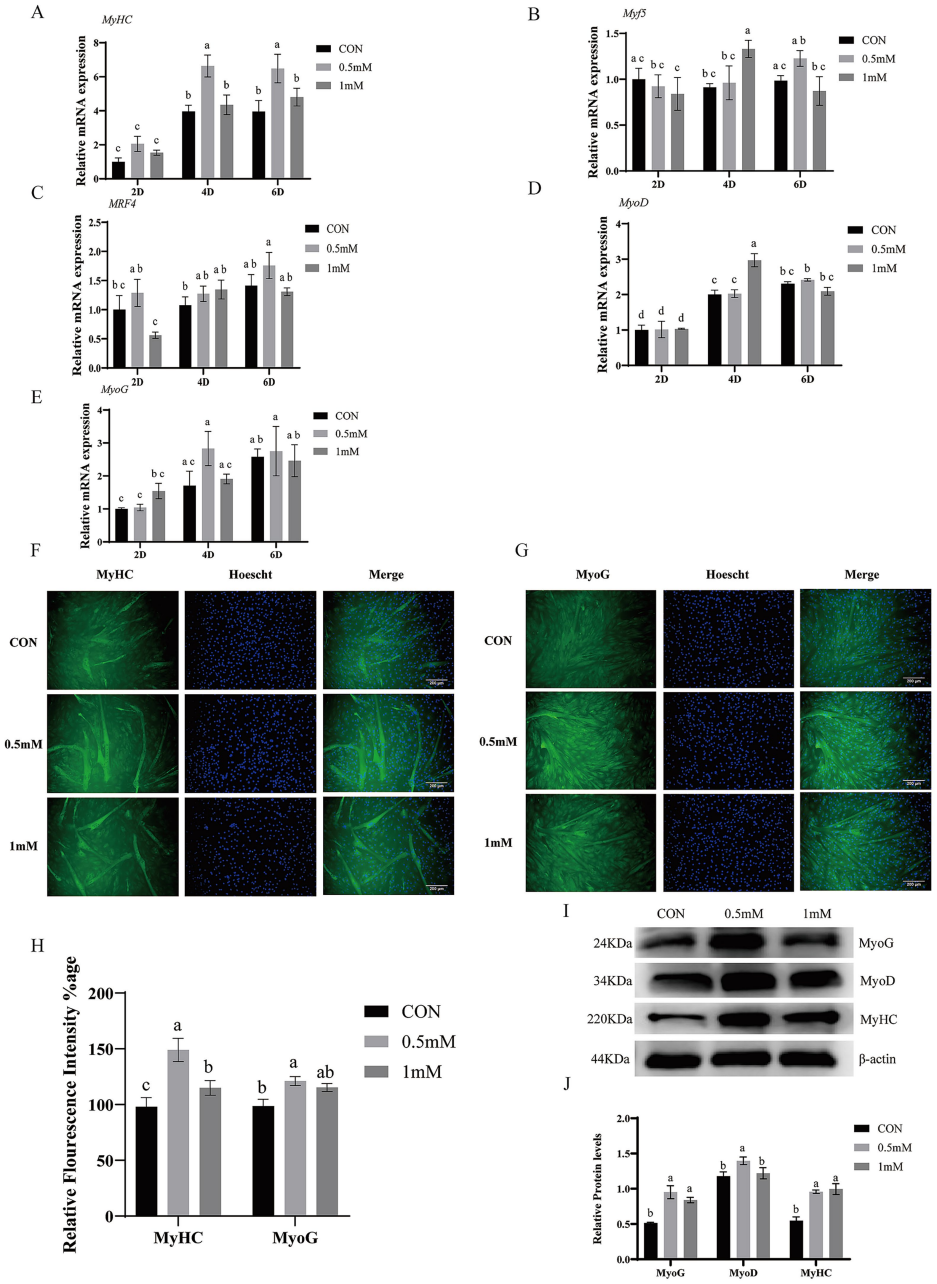
Effects of GABA on myogenic differentiation of BSCs. **(A–E)** RT-qPCR of *MyHC*, *Myf5*, *MRF4*, *MyoD*, and *MyoG* mRNA following 2, 4, and 6 d treatment with 0.5- and 1-mM GABA. **(F,G)** Representative immunofluorescence staining images of MyHC and MyoG after 4 days of differentiation with 0.5- and 1-mM GABA (scale bar = 200 μm). (H) Quantification of fluorescence intensity (ImageJ). **(I,J)** Western blotting of MyoG, MyoD, and MyHC (β-actin control). (Mean ± SD; *n* = 3; groups with different letters a–e differs at *p* < 0.05).

We measured mRNA expression of differentiation related genes after 2-, 4-, and 6-days treatment with 0.5 mM and 1 mM GABA (Figures 2A–E). The 0.5 mM group showed significantly increased expression of *MyHC* mRNA after 2, 4, and 6 days (*p* < 0.05). The mRNA expression of *Myf5* and *MyoD* was significantly increased by 1 mM group at 4d (*p* < 0.05). *MRF4* mRNA expression was significantly upregulated by 0.5 mM group after 6-day treatment (*p* < 0.05), while 1 mM showed no significant difference at 2 and 4 days as compared to control group. The mRNA expression of *MyoG* tends to increase by 0.5 mM group after 4 and 6 days but not significantly different as compared to other groups (*p* > 0.05).

Immunofluorescence staining after 4 days of GABA treatment (Figures 2F–H; quantified in Figure 2H) showed that both the 0.5 mM and 1 mM GABA groups exhibited significantly higher MyHC antibody fluorescence intensity than the control group (*p* < 0.05), with the 0.5 mM group higher than the 1 mM group (*p* < 0.05). MyoG antibody fluorescence was significantly higher in the 0.5 mM group than in the control group (*p* < 0.05), whereas the 1 mM group showed no significant difference compared with the other groups (*p* > 0.05). Subsequently, western blots results (Figures 2I,J) showed that, both the 0.5 mM and 1 mM group significantly increased MyoG and MyHC proteins expression as compared with the control group (*p* < 0.05). MyoD was significantly increased in the 0.5 mM group (*p* < 0.05) but showed no significant change in the 1 mM group (*p* > 0.05) as compared to control.

### 3.6 Effects of GABA on BSC viability under cold/heat stress

**Figure 3:**
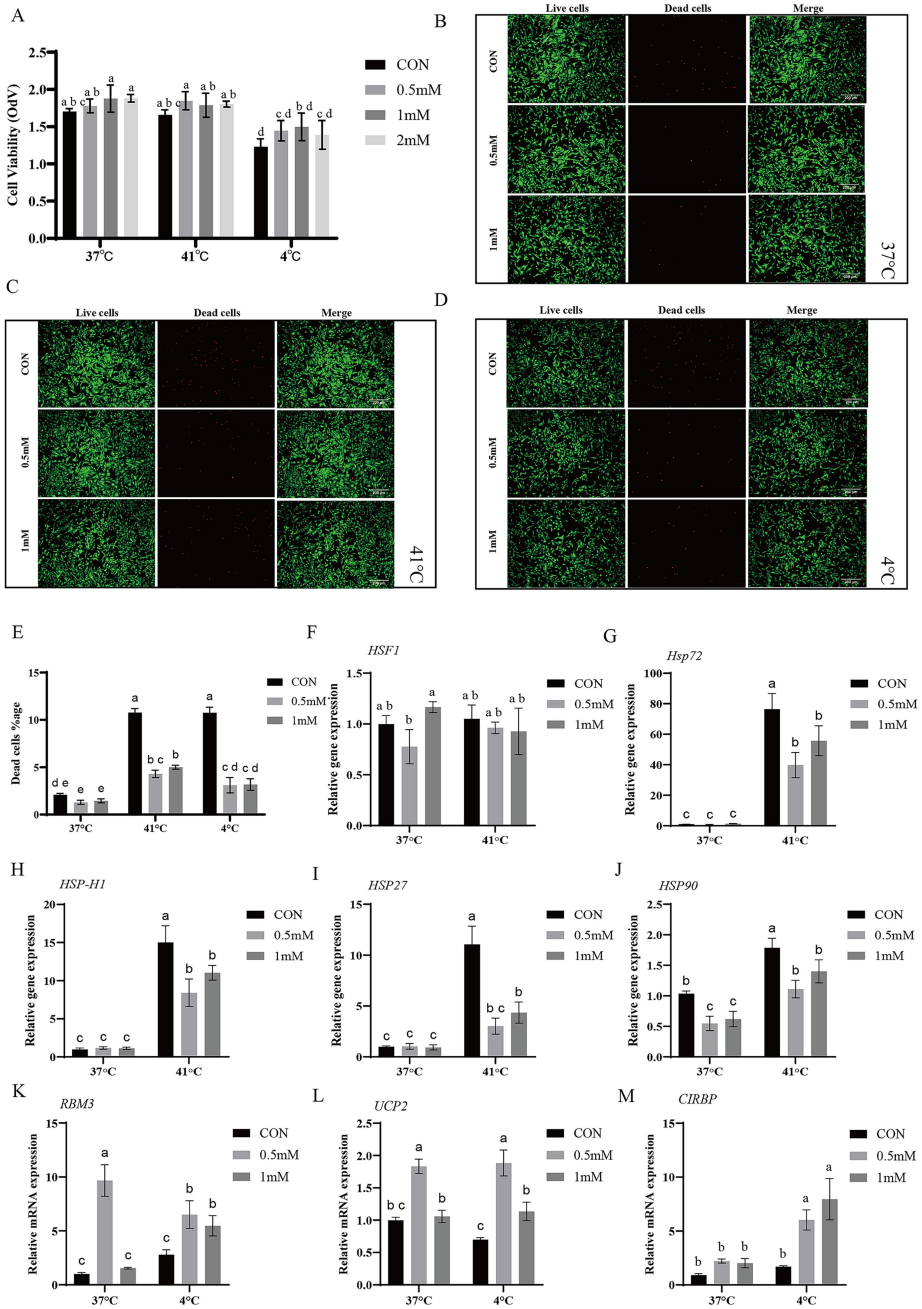
Effects of GABA on BSCs viability and heat/cold-stress-related gene expression. BSCs were pre-treated with 0.5- and 1-mM GABA for 48 h and 6 h placed at 37 °C, 41 °C, or 4 °C. **(A)** CCK-8. **(B–E)** Live/dead staining and percentage of dead cells. **(F–J)** RT-qPCR of heat-stress-related genes mRNA expression (*HSF1*, *Hsp72*, *HSPH1*, *HSP27*, *HSP90*) at 41 °C. **(K–M)** RT-qPCR of cold-stress-related genes mRNA expression (*RBM3*, *UCP2*, *CIRBP*) at 4 °C. (Mean ± SD; *n* = 3; groups with different letters a–d differs at *p* < 0.05).

To examine GABA’s effects under heat/cold stress, we assessed viability by CCK-8 (Figure 3A). Compared with the control group, no significant differences were observed among groups at each temperature (*p* > 0.05). Overall cell viability of control group at 4 °C was decreased as compared to groups at 41 °C and 37 °C (*p* < 0.05).

Live/dead staining (Figures 3B–E) showed no significant differences among groups at 37 °C (*p* > 0.05). Under 41 °C and 4 °C the 0.5 mM and 1 mM group both showed similar significantly lower percentage of dead cells as compared with the control group (*p* < 0.05), indicating protective effects under both heat and cold.

### 3.7 Effects of GABA on heat/cold stress-related mRNA expression

Heat stress-related genes mRNA expressions (Figures 3F–J) were examined. The mRNA expression of *HSF1* showed no significant difference at each treatment between groups. *HSP90* mRNA expression was significantly reduced by GABA treatment as compared to respective control group at both 41 °C and 37 °C (*p* < 0.05). The mRNA expression of *Hsp72*, *HSPH1*, *HSP27* was significantly increased by heat stress as compared to groups at 37 °C, while at 41 °C GABA (0.5 mM and 1 mM) treatment significantly decreased expression as compared to control group (*p* < 0.05). At 37 °C the 0.5 mM GABA group showed significantly higher *RBM3* and *UCP2* mRNA as compared with the control group (*p* < 0.05) (Figures 3K–M). At 4 °C, compared with the control group, all GABA groups significantly increased *RBM3*, *UCP2*, and *CIRBP* mRNA (*p* < 0.05), and *UCP2* mRNA expression was significantly higher in the 0.5 mM group than in the 1 mM group (*p* < 0.05).

### 3.8 Effects of GABA on antioxidant capacity under heat/cold stress

**Figure 4:**
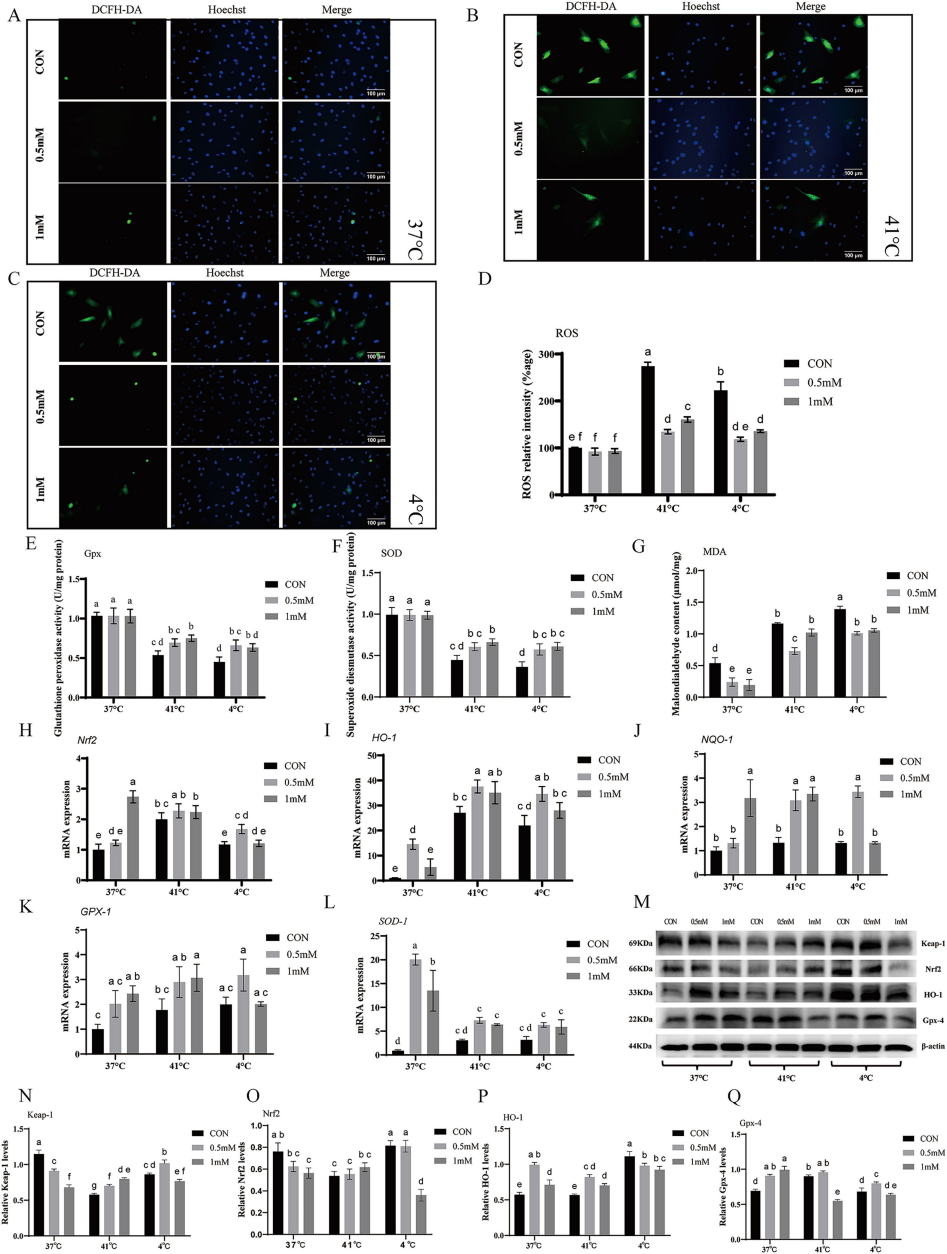
Effects of GABA on oxidative stress and Keap1/Nrf2/HO-1 pathway-related genes and proteins under heat and cold stress. **(A–D)** DCFH-DA detection for ROS after 6 h at 37 °C, 41 °C, and 4 °C; **(E–G)** Antioxidant indices (Gpx, SOD activities; MDA content). **(H–L)** RT-qPCR of *Nrf2*, *HO-1*, *NQO-1*, *GPX-1*, *SOD-1* mRNA. **(M–Q)** Western blotting of Keap-1, Nrf2, HO-1, and Gpx-4 proteins. (Mean ± SD; *n* = 3; groups with different letters a–f differs at *p* < 0.05).

Under the same pretreatments and temperature challenges for 6 h, intracellular ROS were assessed (Figures 4A–D). There were no significant differences among groups at 37 °C as compared with the control group (*p* > 0.05). After heat stress (41 °C) treatment ROS rose sharply in the control group, while the 0.5 mM and 1 mM groups showed significantly reduced ROS accumulation (*p* < 0.05), lower in 0.5 mM group than the 1 mM group (*p* < 0.05). After 4 °C treatment the 0.5 mM and 1 mM GABA group both showed significantly reduced ROS as compared with the control group (*p* < 0.05).

To further evaluate oxidative stress protection (Figures 4E–G), we measured SOD and GPx activities and MDA content. At 37 °C all GABA groups significantly reduced MDA as compared with the control group (*p* < 0.05), while at 41 °C and 4 °C MDA content was significantly decreased by GABA treated groups as compared to their respective control group (*p* < 0.05). The SOD and Gpx enzymes activities showed no significant difference between groups at 37 °C (*p* > 0.05). The 0.5 mM group increased Gpx and SOD activity at 4 °C, while at 41 °C significantly increased by 1 mM as compared to respective control (*p* < 0.05).

### 3.9 Effects of GABA on Keap-1/Nrf2/HO-1 pathway regulation

The Keap1/Nrf2/HO-1 pathway is a key intracellular oxidative stress defense axis that safeguards cellular homeostasis and limits oxidative and inflammatory damage. In this pathway, Keap-1 negatively regulates Nrf2, and Nrf2 controls the transcription of antioxidant genes including HO-1 and NQO-1; Gpx-4 also contributes to redox homeostasis by limiting lipid peroxidation (14, 15).we therefore measured the expression of its core genes and proteins expression. The mRNA expression of *Nrf2*, *NQO-1*, and *GPX-1* genes was upregulated by 1 mM group, while *HO-1* and *SOD-1* mRNA expression was significantly upregulated by 0.5 mM group as compared to control group at 37 °C (*p* < 0.05) (Figures 4H–L). At 41 °C all GABA groups significantly upregulated *NQO-1* mRNA as compared with the control group (*p* < 0.05). *HO-1* and *GPX-1* genes mRNA expressions were significantly increased by 0.5 mM and 1 mM, respectively, as compared to control group (*p* < 0.05). At 4 °C, compared with the control group, the 0.5 mM GABA group significantly upregulated *Nrf2*, *HO-1*, and *NQO-1* mRNA (*p* < 0.05).

Western blots results (Figures 4M–Q) showed that at 37 °C all GABA groups significantly increased HO-1 and Gpx-4 proteins as compared to control group (*p* < 0.05) and significantly decreased Keap-1 (*p* < 0.05), while the 1 mM group showed a significant decrease in Nrf2 (*p* < 0.05). At 41 °C all GABA groups significantly increased Keap-1 and HO-1 proteins as compared with the control group (*p* < 0.05), while the 1 mM group showed a significant decrease in Gpx-4 (*p* < 0.05). At 4 °C all GABA groups significantly decreased HO-1 protein (*p* < 0.05) as compared with the control group; the 0.5 mM group significantly increased Keap-1 and Gpx-4 (*p* < 0.05). Notably, under 4 °C stress, HO-1 mRNA induction did not translate into increased HO-1 protein abundance, indicating a discordance between transcript and protein levels.

### 3.10 Transcriptomic analysis of BSCs under GABA during heat/cold stress

**Figure 5:**
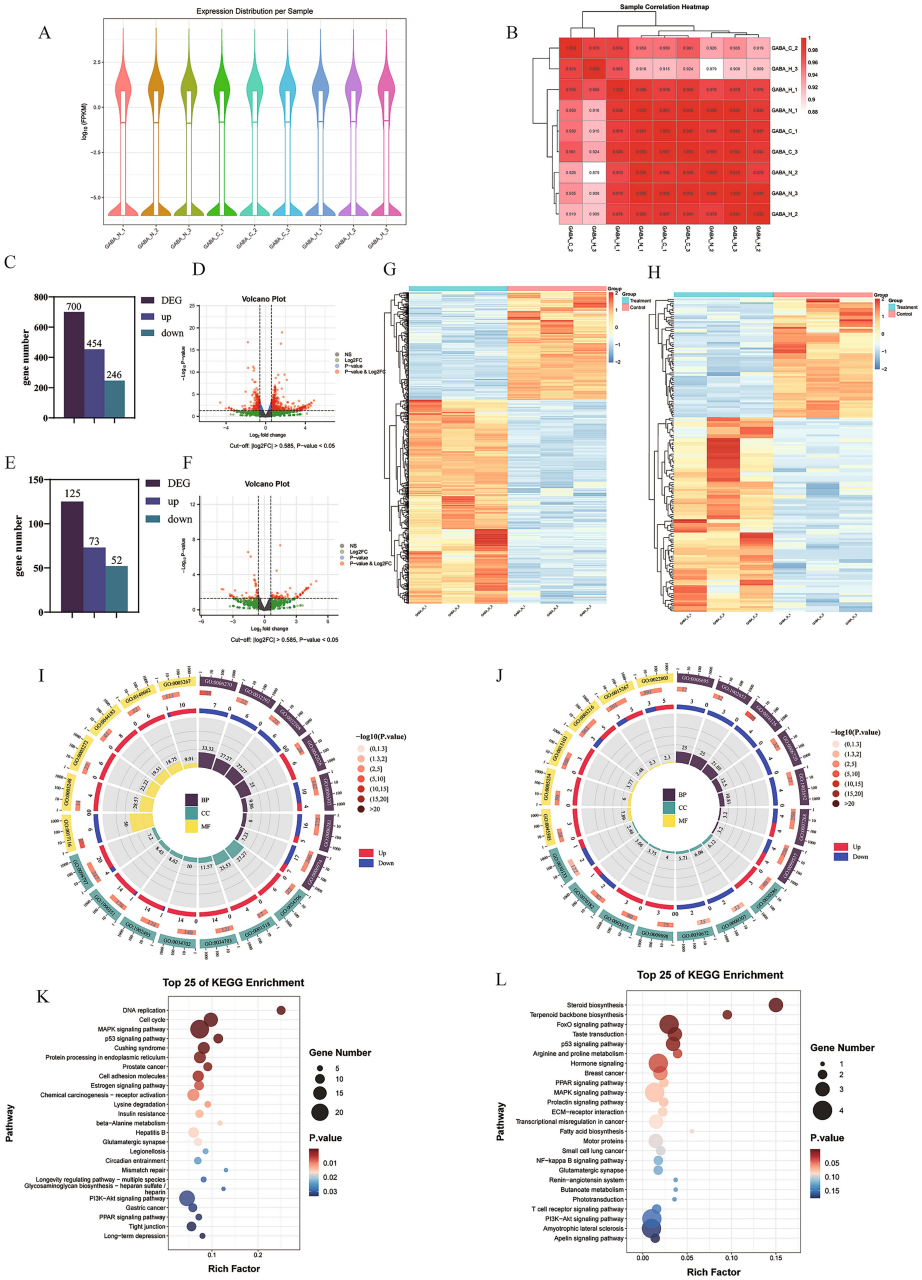
Transcriptomic analysis of BSCs under cold/heat stress with GABA treatment. **(A)** Violin plot of gene expression; **(B)** boxplot of FPKM distributions; **(C,D)** volcano plot of DEGs and bar chart of DEG counts for the 37 °C vs. 41 °C GABA groups, respectively; **(E,F)** volcano plots of DEGs for the 37 °C vs. 4 °C GABA groups; **(G,H)** DEG clustering heatmaps for the 37 °C vs. 41 °C GABA groups and the 37 °C vs. 4 °C GABA groups, respectively; **(I,J)** GO enrichment plots for the 37 °C vs. 41 °C GABA groups and the 37 °C vs. 4 °C GABA groups, respectively; **(K,L)** KEGG pathway enrichment plots showing the Top 25 pathways for the 37 °C vs. 41 °C GABA groups and for the 37 °C vs. 4 °C GABA groups, respectively. GABA_N, GABA_H, and GABA_C denote the 0.5 mM GABA groups at 37 °C, 41 °C, and 4 °C, respectively.

Building on our previous findings, GABA markedly improved the viability, proliferative and differentiation capacity, and survival of BSCs under high (41 °C) and low (4 °C) temperature stress. Notably, there was essentially no significant difference between 0.5 mM and 1.0 mM GABA, indicating that the protective effect had already plateaued at 0.5 mM; therefore, 0.5 mM GABA was used in the subsequent experiments. RNA-seq libraries were constructed. FPKM distributions (Figure 5A) were comparable among groups, and correlation analyses (Figure 5B) indicated strong correlations. Between 37 °C and 41 °C GABA groups, 700 DEGs were identified (454 upregulated; 246 downregulated; Figures 5C,D). Between 37 °C and 4 °C GABA groups, 125 DEGs were identified (73 up; 52 down; Figures 5E,F). Heatmaps showed distinct clustering patterns (Figures 5G,H) GO and KEGG analyses were then performed. For the 37 °C vs. 41 °C GABA groups, GO analysis (Figure 5I; Supplementary Table S3) indicated enrichment of BP terms such as DNA replication initiation and protein refolding, CC terms such as cation channel complex and sodium channel complex, and MF terms such as single stranded DNA helicase activity and protein folding chaperone activity, among others. KEGG analysis (Figure 5K) showed significant enrichment of several oxidative stress-related pathways, including the MAPK, p53, PI3K-Akt and PPAR signaling pathways, among others. It should be noted that pathway enrichment reflects associations based on differential expression patterns and does not by itself demonstrate direct pathway activation. For the 37 °C vs. 4 °C GABA groups, GO analysis (Figure 5J; Supplementary Table S4) revealed enrichment of BP terms such as sterol biosynthetic process and isoprenoid metabolic process, CC terms such as dynein complex and microtubule associated complex, and MF terms such as monovalent ion channel activity and channel activity, among others. KEGG analysis (Figure 5L) further identified oxidative stress-related pathways, including the FoxO and p53 signaling pathways and several hormones signaling pathways, among others.

**Figure 6:**
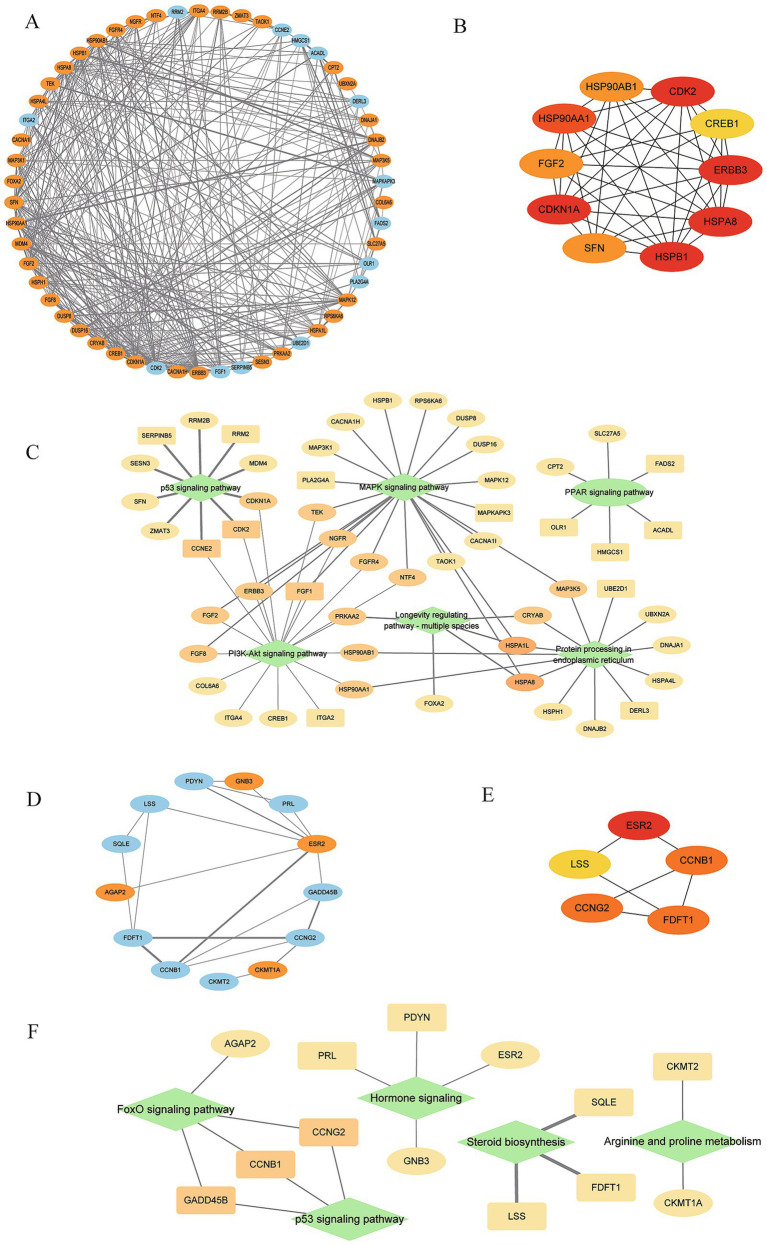
PPI and pathway integration of DEGs under temperature stress. **(A,D)** PPI networks for 37 °C vs. 41 °C and 37 °C vs. 4 °C GABA groups (nodes = proteins; upregulated = orange-red; downregulated = light blue; thicker edges = stronger associations). **(B,E)** Hub nodes (degree metric). **(C,F)** KEGG pathway-gene association networks (diamonds = pathways; ellipses = downregulated; squares = upregulated).

To identify key genes associated with cold and heat stress, we focused on oxidative stress-related pathways in the comparisons between the 37 °C and 41 °C GABA groups and between the 37 °C and 4 °C GABA groups. In total, 53 DEGs were identified between the 37 °C and 41 °C GABA groups, and 13 DEGs were identified between the 37 °C and 4 °C GABA groups. After removing duplicate DEGs, protein–protein interaction (PPI) network analyses were performed separately for the two comparisons (Figures 6A,D). Based on the PPI networks, we used the CytoHubba plugin in Cytoscape to identify hub genes in the network graphs. DEGREE was used as the scoring method to select the top 10 and top 5 hub DEGs for the two comparisons, respectively (Figures 6B,E). Between the 37 °C GABA group and the 41 °C GABA group, the key genes were: Erb-B2 Receptor Tyrosine Kinase 3 (*ERBB3*), *CDK2*, *CDKN1A*, Heat Shock Protein Family B (Small) Member 1 (*HSPB1*), Heat Shock Protein 90 Alpha Family Class A Member 1 (*HSP90AA1*), Heat Shock Protein Family A (Hsp70) Member 8) (*HSPA8*), Fibroblast Growth Factor 2 (*FGF2*), Heat Shock Protein 90 Alpha Family Class B Member 1 (*HSP90AB1*), stratifin (*SFN*), and *CREB1* (cAMP Responsive Element Binding Protein 1). Among these genes, based on gene function and the DEGREE scores (Supplementary Table S5), *CDKN1A* (Degree = 9), *ERBB3* (Degree = 9), *HSPB1* (Degree = 9), and *CDK2* (Degree = 9) were considered central genes closely related to heat induced oxidative stress. Between the 37 °C GABA group and the 4 °C GABA group, the key genes were: *ESR2* (Estrogen Receptor Beta), *FDFT1* (farnesyl-diphosphate farnesyltransferase 1), *CCNG2* (Cyclin G2), *CCNB1* (Cyclin B1), and *LSS* (Lanosterol Synthase). Among these genes, based on gene function and the DEGREE scores (Supplementary Table S6), *ESR2* (Degree = 7), *CCNG2* (Degree = 4), *FDFT1* (Degree = 4), and *CCNB1* (Degree = 4) were considered central genes closely related to cold induced oxidative stress. To clarify the relationships between DEGs and pathways, we constructed DEG-KEGG pathway association networks (Figures 6C,F). In the comparison between the 37 °C and 41 °C GABA groups (Figure 6C), *CDKN1A* and *CDK2* were both enriched in the p53 signaling pathway and the PI3K-Akt signaling pathway. In the comparison between the 37 °C and 4 °C GABA groups (Figure 6F), *CCNG2* and *CCNB* were both enriched in the FoxO signaling pathway and the p53 signaling pathway. Moreover, the p53 signaling pathway was significantly enriched under both heat and cold stress.

### 3.11 Effects of RP-GABA on growth-related measures under different THI conditions

As shown in Supplementary Figure S2, compared with the control group, RP-GABA supplementation showed a trend toward increased body weight under THI 45, THI 63, and THI 28; however, the differences between groups within each THI condition were not significant (*p* > 0.05). As shown in Supplementary Figure S3, RP-GABA supplementation affected selected growth-related morphometric indices. Compared with the control group, body length was significantly increased in the RP-GABA group at THI 45 and THI 63 (*p* < 0.05), whereas no significant difference was observed at THI 28 (*p* > 0.05). For the remaining morphometric measurements, including body height, hip width, hip bone, chest width, chest depth, cannon bone, ischium width, and heart girth, there were no significant differences between the RP-GABA and control groups within each THI condition (*p* > 0.05).

## 4 Discussion

In this study, we show that GABA promotes the proliferation and differentiation of BSCs and improves their resistance to heat and cold stress. We next discuss the potential mechanisms involved. In this study, compared with the control group, the GABA groups showed a significantly higher proportion of EdU-positive BSCs, consistent with CCK-8 results and supporting GABA’s pro-proliferative effect. *PCNA* is the replication fork “sliding clamp” that coordinates polymerases to complete S-phase DNA synthesis and repair; elevated levels indicate enhanced replication (16). *Ki-67* is expressed in G1, S, G2, and M phases but absent in G0 and is widely used as a “growth-fraction” marker (17). *CCND1* drives early G1 progression; *CDK2* is a critical threonine kinase essential for satellite cell proliferation (18). *Pax7* and *Pax3* maintain satellite-cell self-renewal and expansion (19). In this study, both 0.5 mM and 1 mM GABA increased *PCNA*, *Ki-67*, *CCND1*, *Pax3*, and *Pax7* mRNA and elevated PAX7, CDK1, and CDK2 protein levels, suggesting enhanced proliferative potential. Mechanistically, GABA can signal through GABA_A/GABA_B receptors to trigger Ca^2+^-dependent pathways and activate PI3K-Akt/CREB-IRS2 survival/proliferation signaling, thereby promoting BSC proliferation and survival (20). Cyclin D-CDK4/6 drives G1 progression, while Cyclin E-CDK2 controls the G1/S transition and initiates DNA replication (21). Therefore, PI3K-Akt/CREB signaling may contribute to the upregulation of the *CDK2*/*CCND1* axis and proliferation markers such as *PCNA* and *Ki-67* observed in this study. *MyoD* and *Myf5* commit cells to the myogenic lineage and drive early differentiation; *MyHC* marks mature myotubes/myofibers (22, 23). Studies have shown that GABA increases *MyoD* expression in myogenic cells, enhances myotube formation and myosin levels, promotes cell fusion, and elevates *MyHC* levels, indicating that it possesses promyogenic differentiation activity (7, 24). Consistently, 1 mM GABA increased *MyoD* mRNA, whereas 0.5 mM GABA increased *MyHC* mRNA and protein levels. In addition, 1 mM GABA increased *Myf5* and *MyoD* mRNA, supporting a role for GABA in myogenic differentiation. In Ca^2+^-dependent myogenesis, GABA may activate calcineurin-NFAT and CaMK-MEF2, cooperate with *MyoD* to relieve HDAC repression, upregulate *MyoG* and sarcomeric genes, and promote differentiation/fusion (25, 26).

Heat shock proteins (HSPs) are conserved stress proteins that rise in response to heat, oxidative, physical, or chemical stressors (27). Compared with 37 °C, heat stress at 41 °C significantly increased *Hsp72*, *HSP-H1*, *HSP27*, and *HSP90* mRNA expression, consistent with a typical heat-shock response. Under 41 °C heat stress, all concentrations of GABA significantly downregulated the mRNA expression of *Hsp72*, *HSP-H1*, *HSP27*, and *HSP90* in BSCs, indicating that GABA may alleviate protein misfolding pressure induced by high temperature, thereby reducing the magnitude of HSPs transcriptional induction (28, 29). Under 4 °C cold stress, GABA markedly increased the expression of *RBM3*, *CIRBP*, and *UCP2*. *RBM3* and *CIRBP* are cold induced RNA binding proteins that stabilize target mRNAs and promote translation, thus maintaining the synthesis of key proteins and cell survival when low temperature suppresses translation (30, 31). Upregulation of *UCP2* can reduce mitochondrial superoxide generation via mild uncoupling, thereby lowering ROS accumulation in BSCs and the consequent lipid peroxidation and protein damage (32, 33). Heat and cold stress can disrupt redox homeostasis and induce oxidative stress. In this state, excessive ROS elicit lipid peroxidation, oxidative modification/base oxidation/strand breakage of DNA, and protein oxidation, thereby triggering cell cycle arrest, apoptosis, or necrosis (34). In this study, under both heat and cold stress, all concentrations of GABA significantly reduced ROS, indicating that GABA may lower ROS via endogenous antioxidant mechanisms: first, by activating the Nrf2-ARE axis to increase the expression of phase II detoxifying/antioxidant enzymes (14); and second, by cooperating with PI3K-Akt/CREB survival signaling and suppression of mitochondrial ROS to reduce oxidative burden and ROS accumulation (20). Gpx uses GSH as a reductant to catalytically remove H_2_O_2_ and organic peroxides (35), SOD is a primary defense enzyme against radical damage, eliminating the oxygen radical that precedes H_2_O_2_ and hydroxyl ion formation and protecting cells from toxic oxygen free radicals (36). MDA is a degradation product of polyunsaturated fatty acid oxidation and indicates the extent of lipid peroxidation mediated by oxygen free radicals (37). Studies have shown that gavage with GABA significantly increases Gpx and SOD activities in the gastrocnemius muscle of high fat diet mice (38). Dietary GABA enhances SOD and Gpx activities in chicken serum and liver and reduces MDA content (11). Consistent with these findings, in the present study GABA significantly reduced MDA and significantly increased Gpx and SOD activities under heat/cold stress. Beyond acting as an antioxidant to scavenge some ROS itself, more importantly, GABA functions as a signaling molecule that activates pathways such as Nrf2 to upregulate the expression and activity of antioxidant enzymes including SOD and Gpx, thereby systemically strengthening the overall cellular antioxidant defense (38). Furthermore, at 37 °C, 0.5 mM and 1 mM GABA significantly upregulated HO-1 and GPx-4 protein levels and suppressed Keap-1, indicating that GABA can activate antioxidant response element (ARE) driven gene transcription by relieving Keap-1-mediated inhibition of Nrf2 (15). At 41 °C, 0.5 mM GABA significantly induced HO-1 protein expression. HO-1 is a member of the heat-shock protein family, an oxidative stress marker that supports iron homeostasis, antioxidant defense, and prevention of apoptosis (39). It has been reported that under heat stress (40 °C and 42 °C), HO-1 protein expression is significantly increased in bovine granulosa cells (39), Our findings are broadly consistent with the above study. Under heat stress, GABA markedly increased HO-1 protein expression; however, Keap-1 protein was also elevated. We therefore speculate that the increase in HO-1 under heat is more likely driven primarily by heat induced HSF1-NRF2 cross-talk at key regulatory elements (such as E1 and E2) in the HMOX1 promoter, rather than solely by a Keap-1-mediated reduction in NRF2 repression. The precise role and relative importance of this mechanism compared with Keap-1-dependent regulation require further experimental validation (40). At 4 °C, 0.5 mM GABA markedly increased *Nrf2*, *HO-1*, and *NQO-1* mRNA expression, but HO-1 protein levels significantly decreased; the 1 mM GABA group also showed a significant decrease. Studies in renal epithelial cells indicate that under antioxidant stimulation (e.g., curcumin), when temperature drops from 37 °C to 10 °C, HO-1 protein expression decreases and becomes undetectable as the temperature falls further (41). Given that multiple factors affect HO-1 stability, the decline of HO-1 protein observed here may result from proteasomal degradation at low temperature (42, 43). Notably, under 4 °C stress, *HO-1* mRNA increased whereas HO-1 protein decreased, suggesting post-transcriptional regulation; accordingly, the antioxidant and cytoprotective effects of GABA at 4 °C are better supported by reduced ROS/MDA and induction of *RBM3*/*CIRBP*/*UCP2* and *GPx*/*SOD*.

To elucidate the differential mechanisms by which GABA mitigates oxidative stress under heat and cold, transcriptomic analysis revealed that, compared with the 37 °C group, 53 DEGs were identified in the 41 °C high temperature group, among which *CDKN1A*, *ERBB3*, *HSPB1* (Heat Shock Protein Family B (Small) Member 1), and *CDK2* were hub genes in the PPI network. *CDKN1A* (p21), a key effector in the p53 pathway, binds Cyclin-CDK complexes to inhibit CDK2 activity, arresting the cell cycle at G1/S and allowing time for DNA repair (44, 45). In this study, heat upregulated *CDKN1A* while suppressing *CDK2*, consistent with the involvement of p53-associated cell-cycle control and a *CDKN1A* mediated checkpoint that may limit ROS-related damage under high temperature (46). *HSPB1*, a crucial member of the small heat shock protein family, stabilizes the cytoskeleton (e.g., actin filaments), inhibits ROS generation, reduces accumulation of lipid peroxidation products, and modulates apoptotic signaling (47, 48). In this study, *HSPB1* was significantly upregulated under heat and served as a core node in the PPI network, suggesting it may be a key effector through which GABA helps muscle cells cope with high temperature. For 4 °C cold stress, compared with the 37 °C GABA group, 13 DEGs were identified in the 4 °C group, among which *ESR2*, *CCNB1*, *CCNG2*, and *FDFT1* were hub genes. *ESR2* (also ERβ) is a nuclear estrogen receptor that regulates gene expression upon estrogen binding and plays important roles in reproductive and nonreproductive systems (49). In bovine ovarian cysts, *ESR2* mRNA expression is significantly downregulated during cyst formation (50). In our experiment, cold stress significantly upregulated *ESR2*; the specific mechanism requires further study. *CCNB1* is a key regulator of the G2/M transition; *CCNB1* forms a complex with *CDK1* to drive entry into mitosis. Under cold stress in our study, GABA may involve p53-associated signaling and may downregulate *CCNB1*, thereby limiting entry into M phase and reducing chromosome segregation errors under low temperature stress (51). *CCNG2* is a typical cyclin that is induced in growth inhibitory contexts and can form an active complex with protein phosphatase PP2A, acting as a negative regulator to suppress cell cycle progression (52, 53). In our cold stress condition with GABA treatment, *CCNG2* was downregulated; *CCNG2* transcription is positively regulated by FoxO, whereas activation of PI3K-Akt suppresses FoxO transcriptional activity via phosphorylation, thereby reducing *CCNG2* expression (54). We found that the p53 signaling pathway was significantly enriched under both high and low temperature stress. Under heat, the data were consistent with p53-associated signaling and, with inducing *CDKN1A* and suppressing *CDK2*, arrested the cell cycle at G1/S to allow time for DNA repair (44, 45); under cold, the data were also consistent with p53-associated regulation, potentially involving transcriptional repression of *CCNB1*, thereby reducing G2-to-M entry (51), together, both scenarios limit replication and division of damaged DNA, thereby protecting cells. These results suggest that p53-associated signaling may represent a shared hub in the BSC response to temperature stress. As these p53/PI3K-Akt/FoxO findings are based on enrichment analyses, they should be interpreted as associations rather than evidence of causality. In addition to the BSC based *in vitro* experiments, we included an *in vivo* RP GABA trial under different THI conditions. RP-GABA supplementation showed a trend toward higher body weight and significantly increased body length at THI 45 and THI 63, while most other morphometric measures did not differ significantly. Overall, these in vivo data provide limited supportive evidence that GABA may help maintain growth-related measures under temperature-related challenges; however, muscle quality and muscle tissue endpoints were not assessed and warrant further validation.

## 5 Conclusion

In summary, GABA significantly promotes the proliferation and myogenesis of bovine BSCs and, under 41 °C and 4 °C stress, reduces ROS while enhancing the antioxidant capacities of SOD and Gpx. Mechanistically, GABA increased HO-1 and reduced HSP induction under heat stress; under cold stress it induced *RBM3*/*CIRBP*/*UCP2* and reduced ROS, although *HO-1* mRNA and protein levels were not concordant. Transcriptomics analysis further suggests p53 as a common hub: under heat, GABA’s protective effects relate to PI3K-Akt signaling and genes such as *CDKN1A* and *CDK2*; under cold, GABA works in parallel with FoxO signaling to regulate *CCNB1* and *CCNG2*, limiting entry of damaged cells into division. These transcriptomic associations require further validation to establish causality. Integrating CCK-8, EdU, differentiation markers, antioxidant indices, and ROS, 0.5 mM performed better or not worse than 1 mM at most timepoints and endpoints; thus, under the present conditions, 0.5 mM is recommended as the preferred comprehensive dose and can be prioritized for subsequent applications. In the in vivo RP-GABA trial, RP-GABA significantly increased body length at THI 45 and THI 63. This study provides a molecular basis for developing GABA-based functional feeds to mitigate temperature stress in ruminants and to improve growth performance in beef cattle.

## Data Availability

The data presented in the study are deposited in the NCBI Sequence Read Archive (SRA) repository under BioProject accession number PRJNA1441250 and are publicly available at https://www.ncbi.nlm.nih.gov/sra/PRJNA1441250.

## Footnotes

1
http://string-db.org
