# Supplementary material for: γ-Aminobutyric acid enhances myogenesis and heat/cold stress resistance in bovine muscle satellite cells
Source: Front Vet Sci. 2026 Apr 14;13:1770540. doi: 10.3389/fvets.2026.1770540 (PMC13120942; doi:10.3389/fvets.2026.1770540)
Supplement: Supplementary file 1 [file Table_1.docx]

***Supplementary Material***

| **Supplementary Table S1. Genes and primer sequences** | | | |
| --- | --- | --- | --- |
| **Genes** | **Accession number** | **Sequence（5’–3’）** | **Product size (bp)** |
| *CDK2* | NM_001014934.1 | F: GACGGAGCTTGTTATCGCAAATGC | 108 |
|  |  | R: GAGGTACTGGCTTGGTCACATCTTG |  |
| *PCNA* | NM_001034494.1 | F: GTCCAGGGCTCCATCTTGAAGAAAG | 225 |
|  |  | R: GCTGCACCAAGGAGACATGAGAC |  |
| *Ki67* | XM_015460791.2 | F: CAGTCAACACGCCGACCAGTAAG | 144 |
|  |  | R: CATCACCTGCTGCTTCTCCTTCTG |  |
| *Pax7* | XM_015460690 | F: CTCAGTGAGTTCGATTAGCCG | 180 |
|  |  | R: AGACGGTTCCCTTTGTCGC |  |
| *CCND1* | NM_01034494.1 | F: CTCGGTGTCCTACTTCAAGTGTGTG | 143 |
|  |  | R: TCGCAGACCTCCAGCATCCAG |  |
| *Pax3* | NM_001206818 | F: GGCTGCGTCTCTAAGATCCT | 158 |
|  |  | R: ATTTCCCAGCTGAACATGCC |  |
| *MyHC* | NM_001101835.1 | F: GCTGGCTGGAGAAGAACAAGGAC | 102 |
|  |  | R: TGGTGAAGGTGGCGTAGAGGTG |  |
| *Myf5* | NM_174116 | F: CCCACCTCAAGTTGCTCTGA | 115 |
|  |  | R: CCGTGGCATATACATTTGGTACA |  |
| *MRF4* | NM_181811 | F: TGGACCCCTTCAGCTACAGA | 139 |
|  |  | R: ATGCTTGTCCCTCCTTCCTTG |  |
| *MyoD* | NM_001040478 | F: CCGACGGCATGATGGACTA | 80 |
|  |  | R: CTCGCTGTAGTAAGTGCGGT |  |
| *MyoG* | NM_001111325 | F: CAGTGAATGCAGCTCCCATAG | 87 |
|  |  | R: GCAGATGATCCCCTGGGTTG |  |
| *HSF1* | NM 001076809.1 | F: CCAGCAACAGAAAGTCGTCA | 92 |
|  |  | R: GGGGGATCTTTCTCTTCACC |  |
| *Hsp72* | NM 174344.2 | F: TCGGAAGATGAGGCCAATCG | 102 |
|  |  | R: GCCCCTCAGTTTCTCGTCTI |  |
| *HSP-H1* | NM 001075302.1 | F: AGCCCCAGGTACAAACTGATG | 296 |
|  |  | R: CTCCACCGCATTCTTAGCGT |  |
| *HSP27* | NM 001025569.1 | F: TGAAACACCGCCTGCTAAAAA | 188 |
|  |  | R: GATACCAGTCGCGGAAAGGG |  |
| *HSP90* | NM 001012670.2 | F: TGCCAGGCCTCTAGAAGTTAG | 74 |
|  |  | R: ACAAGCCCGATGTATGGACA |  |
| *RBM3* | NM 001303463.1 | F: GTCCAGAGACTATGGTGGCAG | 137 |
|  |  | R: TCATTTGGAGAGGTGGTCCTG |  |
| *UCP2* | AF127029 | F: GAGCACTGTTGAGGCCTACA | 136 |
|  |  | R: GCCTTCAGGAGAGTGTCCTT |  |
| *CIRBP* | NM 001034278.1 | F: GCTCTGGTCGTTTTGCTCTC | 107 |
|  |  | R: CTCAGCCTCCGACGAAAAG |  |
| *Nrf2* | NM_001011678.2 | F: CCTCAAAGCACCGTCCTCAG | 362 |
|  |  | R: ATCTGCTTTGGGAATGGGGG |  |
| *HO-1* | NM_001014912.1 | F: CAAGCGCTATGTTCAGCGAC | 206 |
|  |  | R: GCTTGAACTTGGTGGCACTG |  |
| *NQO-1* | NM_001034535.1 | F: TTTGAGCGGGTGCTCATAGG | 124 |
|  |  | R: GAGAGTACATGGAGCCGCTG |  |
| *GPX-1* | NM_174076.3 | F: AACGTAGCATCGCTCTGAGG | 121 |
|  |  | R: GATGCCCAAACTGGTTGCAG |  |
| *SOD-1* | NM_174615.2 | F: TTCTCTACTTGGTTGGGGCG | 133 |
|  |  | R: CCCTTTGCCTCGAAGTGGAT |  |

**Supplementary Table S2. Composition and nutrient levels of the experimental diet (dry matter basis)**

| **Item** | **Content** |
| --- | --- |
| **Ingredients, % of DM** |  |
| Corn stover | 45.00 |
| Corn | 37.50 |
| 8330 concentrate feed**^1)^** | 17.50 |
| **Nutrient levels (DM basis)** |  |
| Comprehensive net energy**^2)^**, MJ/kg | 9.31 |
| Crude protein, % | 11.03 |
| Crude fat, % | 5.37 |
| Neutral detergent fiber (NDF), % | 49.53 |
| Acid detergent fiber (ADF), % | 23.32 |
| Crude ash, % | 8.23 |
| Soluble carbohydrates, % | 3.45 |
| Non-fiber carbohydrates (NFC), % | 29.08 |
| **Notes:** 1) 8330 concentrate feed: moisture 14%, crude protein 35%, crude fiber 15%, crude ash 20%, Ca 1%–3%, P 0.5%–1%, NaCl 0.5%–4%, lysine 1.2%.  2) Comprehensive net energy is a calculated value. NFC is calculated as: NFC (% of DM) = 100 − (crude protein + crude fat + crude ash + NDF). Other nutrients are measured values. | |

| **Supplementary Table S3. GO enrichment analysis of DEGs between 37°C and 41°C GABA groups** | | | | |
| --- | --- | --- | --- | --- |
| GO_ID | GO_Term | GO_Category | IDs | *P*.value |
| GO:0006270 | DNA replication initiation | BP | *CDT1, CDC6, MCM3, MCM2, MCM5, MCM4, MCM7* | 1.99E-06 |
| GO:0042026 | protein refolding | BP | *CRYAB, HSPB1, HSPA8, DNAJB2, DNAJA1, LOC782779, HSPA1L* | 1.68E-05 |
| GO:0032392 | DNA geometric change | BP | *MCM3, MCM2, MCM8, MCM5, MCM4, MCM7* | 4.02E-05 |
| GO:0032508 | DNA duplex unwinding | BP | *MCM3, MCM2, MCM8, MCM5, MCM4, MCM7* | 4.02E-05 |
| GO:0006281 | DNA repair | BP | *SLX4, POLE2, PCNA, SLF1, NSMCE2, ALKBH3, RNASEH2A, DCLRE1C, MCM3, REV3L, MCM2, MCM8, MCM5, PRKDC, MCM4, GINS4, RFC2, MLH3, FANCE, UNG, MCM7, RAD51AP1* | 4.35E-05 |
| GO:0006974 | DNA damage response | BP | *RPS6KA6, SLX4, POLE2, PPP4R3A, PCNA, SLF1, NSMCE2, ALKBH3, RNASEH2A, DCLRE1C, MCM3, REV3L, MCM2, MCM8, MCM5, PRKDC, MCM4, GINS4, DTL, RFC2, MLH3, FANCE, UNG, MCM7, RAD51AP1* | 7.18E-05 |
| GO:0006302 | double-strand break repair | BP | *SLX4, SLF1, NSMCE2, DCLRE1C, MCM3, REV3L, MCM2, MCM8, MCM5, PRKDC, MCM4, GINS4, MCM7, RAD51AP1* | 0.000123238 |
| GO:0034703 | cation channel complex | CC | *SCN9A, KCNG1, KCNC1, RYR1, GRIK3, KCNG2, UNC80, GRIK4, KCNIP3, SCN3A, KCNA4, CACNA1H, KCNG3, CACNA1I* | 3.09E-05 |
| GO:0034706 | sodium channel complex | CC | *SCN9A, GRIK3, GRIK4, SCN3A, CACNA1H, CACNA1I* | 4.91E-05 |
| GO:0098797 | plasma membrane protein complex | CC | *SSPN, GPR156, SCN9A, KCNG1, KCNC1, CDH22, CDH5, ATP1B4, ITGA4, GRIK3, LIN7A, CDH15, CDH6, KCNG2, APC2, BOLA-DMA, GRIK4, KCNIP3, ITGA2, SCN3A, KCNA4, GJA8, CACNA1H, KCNG3, CACNA1I* | 0.000130981 |
| GO:0034702 | monoatomic ion channel complex | CC | *SCN9A, KCNG1, KCNC1, RYR1, GRIK3, KCNG2, UNC80, GRIK4, KCNIP3, SCN3A, KCNA4, CACNA1H, KCNG3, CACNA1I* | 0.000153273 |
| GO:1902495 | transmembrane transporter complex | CC | *SCN9A, KCNG1, KCNC1, RYR1, ATP1B4, GRIK3, KCNG2, UNC80, GRIK4, KCNIP3, SCN3A, KCNA4, CACNA1H, KCNG3, CACNA1I* | 0.00046037 |
| GO:1990351 | transporter complex | CC | *SCN9A, KCNG1, KCNC1, RYR1, ATP1B4, GRIK3, KCNG2, UNC80, GRIK4, KCNIP3, SCN3A, KCNA4, CACNA1H, KCNG3, CACNA1I* | 0.000584958 |
| GO:0001518 | voltage-gated sodium channel complex | CC | *SCN9A, SCN3A, CACNA1H, CACNA1I* | 0.001744155 |
| GO:0017116 | single-stranded DNA helicase activity | MF | *MCM3, MCM2, MCM8, MCM5, MCM4, MCM7* | 1.02E-06 |
| GO:0044183 | protein folding chaperone | MF | *CRYAB, HSP90AB1, HSPH1, HSP90AA1, HSPB1, HSPA8, HSPA4L, HSPA1L* | 5.11E-05 |
| GO:0005272 | sodium channel activity | MF | *SCN9A, GRIK3, GRIK4, SCN3A, CACNA1H, CACNA1I* | 0.000213185 |
| GO:0140662 | ATP-dependent protein folding chaperone | MF | *HSP90AB1, HSPH1, HSP90AA1, HSPA8, HSPA4L, HSPA1L* | 0.00056688 |
| GO:0005248 | voltage-gated sodium channel activity | MF | *SCN9A, SCN3A, CACNA1H, CACNA1I* | 0.000924148 |
| GO:0005267 | potassium channel activity | MF | *KCNG1, KCNN4, KCNC1, KCNJ3, SNAP25, GRIK3, KCNG2, GRIK4, KCNA4, KCNG3, KCNK9* | 0.001157675 |

| **Supplementary Table S4. GO enrichment analysis of DEGs between 37°C and 4°C GABA groups** | | | | |
| --- | --- | --- | --- | --- |
| GO_ID | GO_Term | GO_Category | IDs | *P*.value |
| GO:0016126 | sterol biosynthetic process | BP | *INSIG1, SQLE, FDFT1, LSS* | 2.18E-06 |
| GO:0006720 | isoprenoid metabolic process | BP | *IDI1, HMGCS1, FDFT1, LSS* | 1.93E-05 |
| GO:0006695 | cholesterol biosynthetic process | BP | *INSIG1, FDFT1, LSS* | 2.62E-05 |
| GO:1902653 | secondary alcohol biosynthetic process | BP | *INSIG1, FDFT1, LSS* | 2.62E-05 |
| GO:1902652 | secondary alcohol metabolic process | BP | *INSIG1, SQLE, FDFT1, LSS* | 3.48E-05 |
| GO:0007268 | chemical synaptic transmission | BP | *RIC3, CADPS, CACNG8, SNAP25, VGF, SLC24A1, PDYN, MCTP1* | 3.67E-05 |
| GO:0099537 | trans-synaptic signaling | BP | *RIC3, CADPS, CACNG8, SNAP25, VGF, SLC24A1, PDYN, MCTP1* | 3.67E-05 |
| GO:0030286 | dynein complex | CC | *DNAH2, DNAH8, DNAH9* | 0.002058172 |
| GO:0009898 | cytoplasmic side of plasma membrane | CC | *AJAP1, ZAP70, GNB3* | 0.006854115 |
| GO:0005875 | microtubule associated complex | CC | *DNAH2, DNAH8, DNAH9* | 0.008187886 |
| GO:0070382 | exocytic vesicle | CC | *SYNGR3, SYTL1, MCTP1* | 0.008761153 |
| GO:0000307 | cyclin-dependent protein kinase holoenzyme complex | CC | *CCNB1, CCNG2* | 0.012539408 |
| GO:0030672 | synaptic vesicle membrane | CC | *SYNGR3, MCTP1* | 0.014037073 |
| GO:0030133 | transport vesicle | CC | *SYNGR3, SYTL1, MCTP1* | 0.025237328 |
| GO:0005216 | monoatomic ion channel activity | MF | *KCNE4, CACNG8, SNAP25, SLC24A3, ANO8, SCN3A, SLC24A1, CLCNKA, ANO9* | 8.39E-05 |
| GO:0015267 | channel activity | MF | *KCNE4, CACNG8, SNAP25, SLC24A3, ANO8, SCN3A, SLC24A1, CLCNKA, ANO9* | 0.00014728 |
| GO:0022803 | passive transmembrane transporter activity | MF | *KCNE4, CACNG8, SNAP25, SLC24A3, ANO8, SCN3A, SLC24A1, CLCNKA, ANO9* | 0.00014728 |
| GO:0045505 | dynein intermediate chain binding | MF | *DNAH2, DNAH8, DNAH9* | 0.000907447 |
| GO:0015103 | inorganic anion transmembrane transporter activity | MF | *SLC26A1, ANO8, CLCNKA, ANO9* | 0.001990124 |
| GO:0005254 | chloride channel activity | MF | *ANO8, CLCNKA, ANO9* | 0.002019084 |

| **Supplementary Table S5. Information on key genes between 37℃ and 41℃ GABA groups** | | | | |
| --- | --- | --- | --- | --- |
| **Gene name** | **Log2(FC)** | **pvalue** | **Degree** | **Type** |
| *CDKN1A* | 0.746023213 | 1.58827E-05 | 9 | up |
| *ERBB3* | 0.924283634 | 0.017779048 | 9 | up |
| *HSPB1* | 0.972094721 | 6.72E-07 | 9 | up |
| *CDK2* | -0.635127931 | 0.006863094 | 9 | down |
| *HSPA8* | 1.022420644 | 2.14E-09 | 9 | up |
| *HSP90AA1* | 0.788807256 | 1.13E-06 | 8 | up |
| *FGF2* | 0.592409415 | 0.004507003 | 7 | up |
| *HSP90AB1* | 0.608764609 | 0.000200852 | 7 | up |
| *SFN* | 3.571049198 | 0.008167127 | 7 | up |
| *CREB1* | 0.732312778 | 0.007585579 | 6 | up |
| Note: The table just lists the top 10 hub genes . | |  |  |  |

| **Supplementary Table S6. Information on key genes between 37℃ and 4℃ GABA groups** | | | | |
| --- | --- | --- | --- | --- |
| **Gene name** | **Log2(FC)** | **pvalue** | **Degree** | **Type** |
| *ESR2* | 3.97266305 | 0.003859025 | 7 | up |
| *CCNG2* | -0.702673273 | 0.035195968 | 4 | down |
| *FDFT1* | -0.865876864 | 0.000985517 | 4 | down |
| *CCNB1* | -0.705617956 | 0.01204679 | 4 | down |
| *LSS* | -0.684842567 | 0.010952681 | 3 | down |
| Note: The table just lists the top 5 hub genes. | |  |  |  |

**Supplementary Figure S1.** Temperature–humidity index (THI) profiles during the in vivo trial. Daily THI values calculated from ambient temperature and relative humidity recorded at 07:00 (THI_M), 14:00 (THI_N), and 22:00 (THI_E) using the NOAA (1976) equation. The x-axis indicates consecutive recording days, and the y-axis indicates THI values. THI 45, THI 63, and THI 28 represent the mean daily THI values (THI_AVE = average of THI_M, THI_N, and THI_E) during the corresponding phases of the trial.


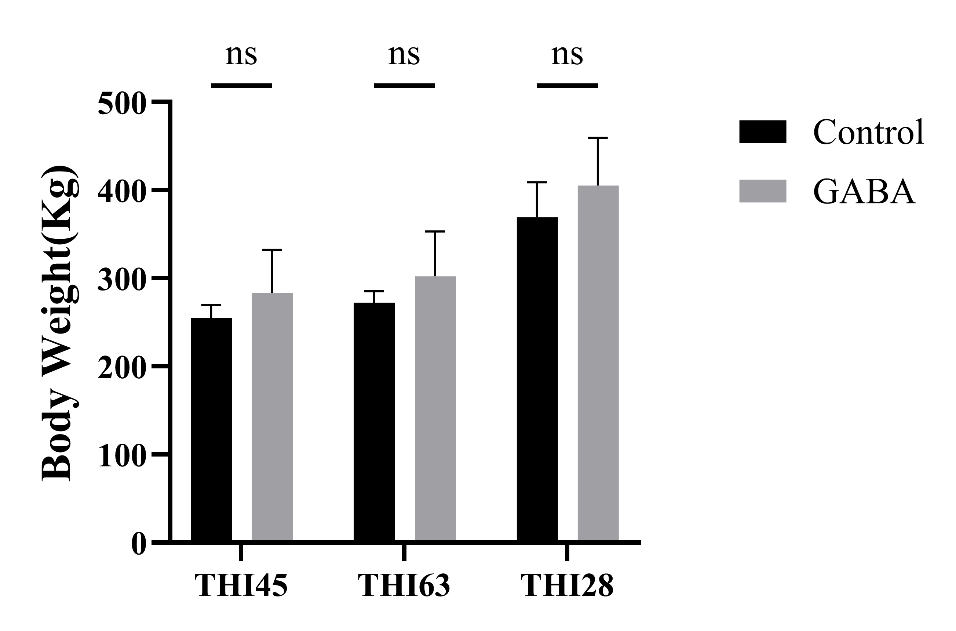


**Supplementary Figure S2.** Effects of RP-GABA on body weight under different THI conditions. Body weight of Yanbian Yellow calves in the control and RP-GABA groups evaluated under THI 45, THI 63, and THI 28 conditions. Data are presented as mean ± SD (n = 7 per group). Statistical comparisons between groups within each THI condition are indicated above the bars (ns, *P* > 0.05).


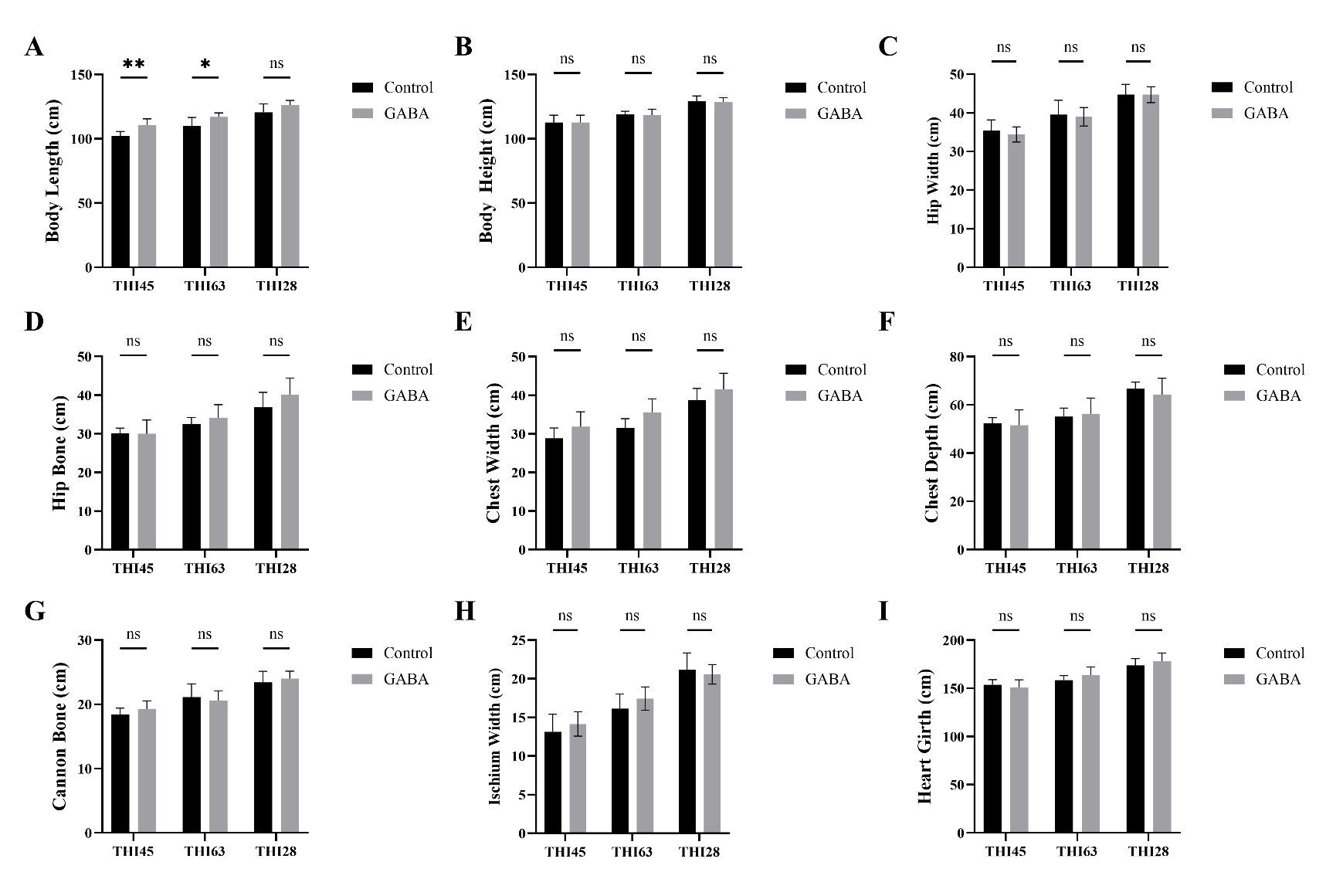


**Supplementary Figure S3.** Effects of RP-GABA on growth-related morphometric indices under different THI conditions. Growth-related morphometric measurements of calves in the control and RP-GABA groups under THI 45, THI 63, and THI 28 conditions, including (A) body length, (B) body height, (C) hip width, (D) hip bone, (E) chest width, (F) chest depth, (G) cannon bone, (H) ischium width, and (I) heart girth. Data are presented as mean ± SD (n = 7 per group). Statistical comparisons between groups within each THI condition are indicated above the bars (* *P* < 0.05; ** *P* < 0.01; ns, *P* > 0.05).
